# Supplementary material for: INPP4B protects from metabolic syndrome and associated disorders
Source: Commun Biol. 2021 Mar 26;4:416. doi: 10.1038/s42003-021-01940-6 (PMC7998001; doi:10.1038/s42003-021-01940-6)

Supplementary Figures and Figure Legends

INPP4B protects from metabolic disease and associated disorders.

Manqi Zhang[1], Yasemin Ceyhan[2], Elena M. Kaftanovskaya[2], Judy L. Vasquez[2], Jean Vacher[3], Filip K. Knop[4,5,6,7], Lubov Nathanson[8], Alexander I. Agoulnik[2,9,10], Michael M. Ittmann[11,12], Irina U. Agoulnik[2,10,13]*

[1]Department of Medicine, Duke University, Durham, NC,

[2]Department of Human and Molecular Genetics, Herbert Wertheim College of Medicine, Florida International University, Miami, FL

[3]Department of Medicine, Institut de Recherches Cliniques de Montréal, Université de Montréal, Montréal, Québec, Canada

[4]Center for Clinical Metabolic Research, Gentofte Hospital, University of Copenhagen, Hellerup, Denmark

[5]Department of Clinical Medicine, Faculty of Health and Medical Sciences, University of Copenhagen, Copenhagen, Denmark

[6]Novo Nordisk Foundation Center for Basic Metabolic Research, Faculty of Health and Medical Sciences, University of Copenhagen, Copenhagen, Denmark

[7]Steno Diabetes Center Copenhagen, Gentofte, Denmark

[8]Institute for Neuro Immune Medicine, Dr. Kiran C. Patel College of Osteopathic Medicine, Nova Southeastern University, Ft. Lauderdale, FL

[9]Department of Obstetrics and Gynecology, Baylor College of Medicine, Houston, TX

[10]Biomolecular Sciences Institute, Florida International University, Miami, FL

[11]Department of Pathology and Immunology, Baylor College of Medicine, Houston, Texas, USA.

[12]Michael E. DeBakey Department of Veterans Affairs Medical Center, Houston, Texas, USA.

[13]Department of Molecular and Cellular Biology, Baylor College of Medicine, Houston, Texas, USA.

***Correspondence:** iagoulni@fiu.edu.

Supplementary figure 1

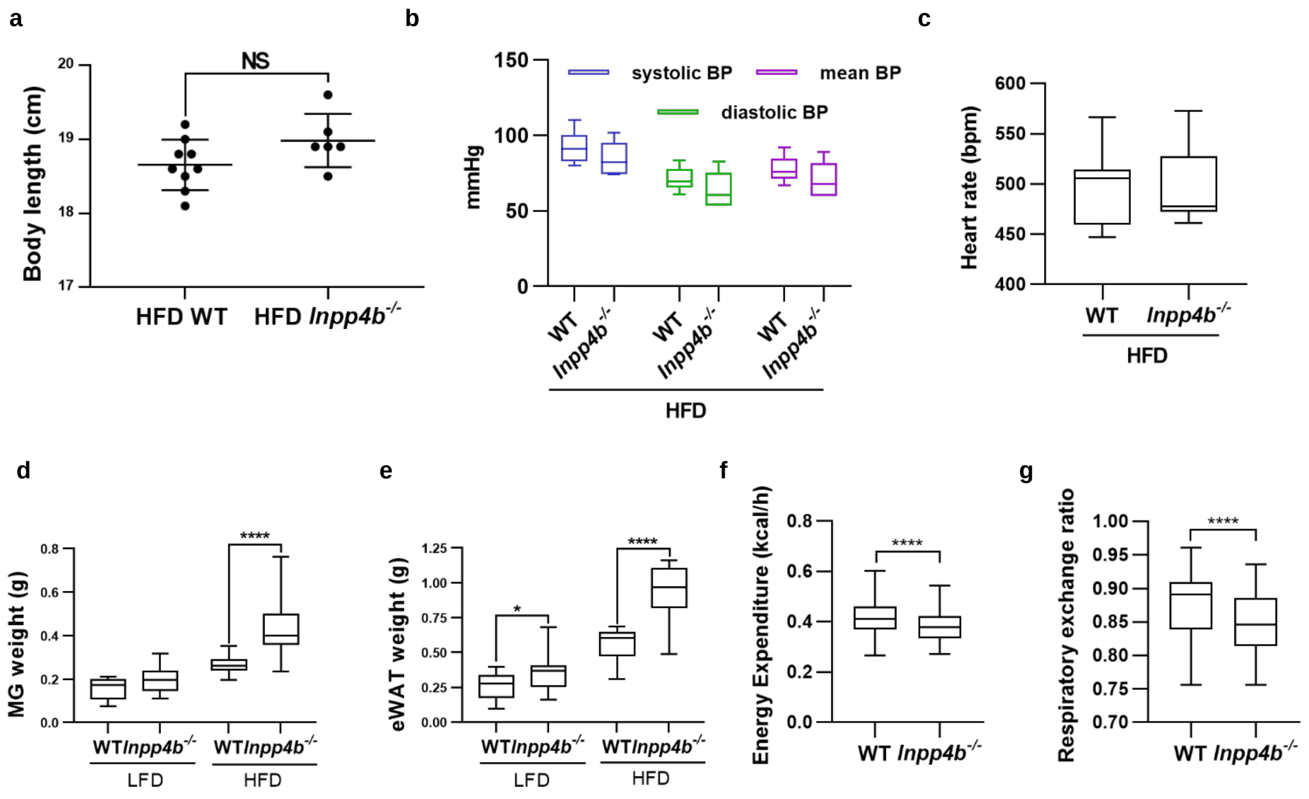

Supplementary figure 2

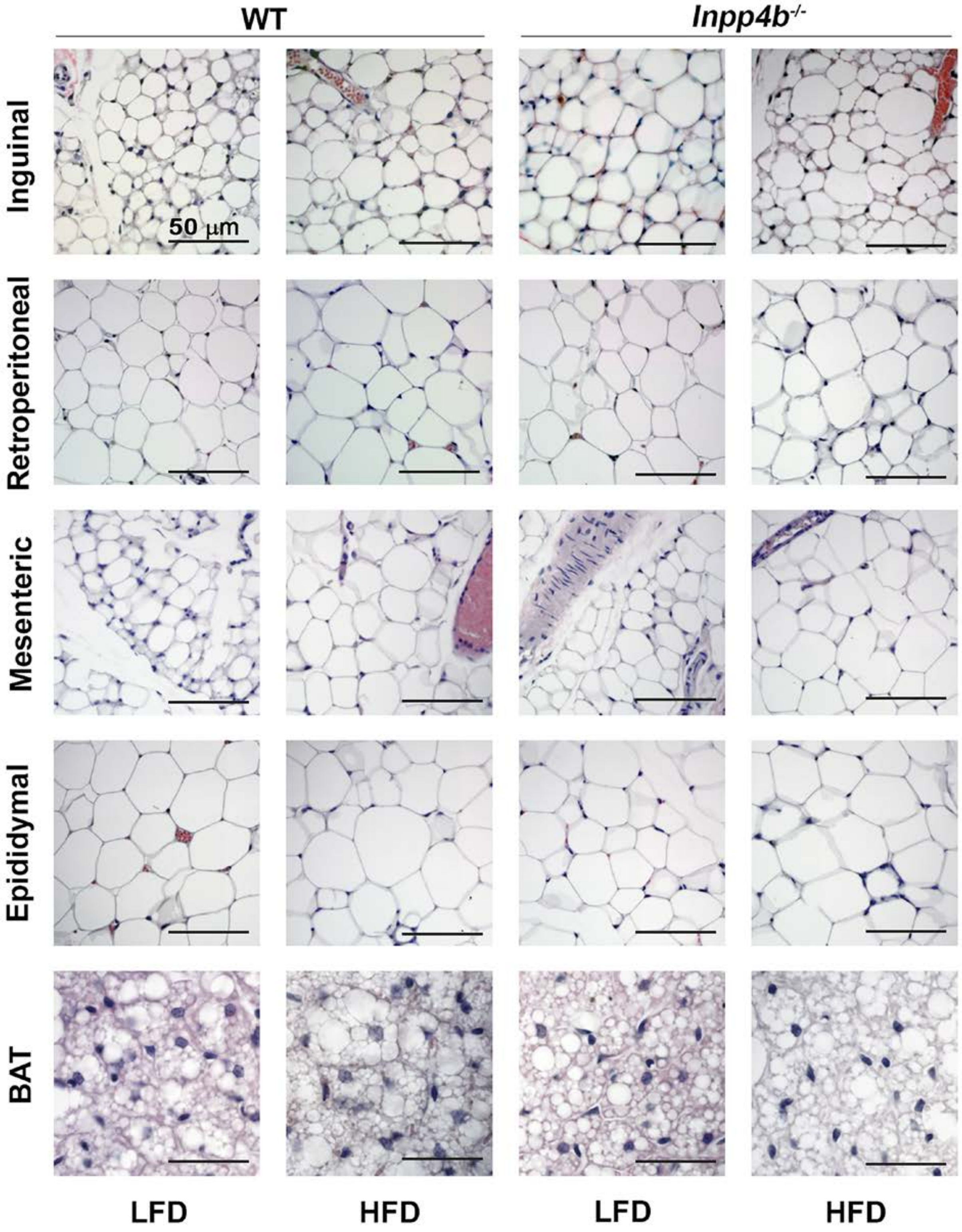

Supplementary figure 3

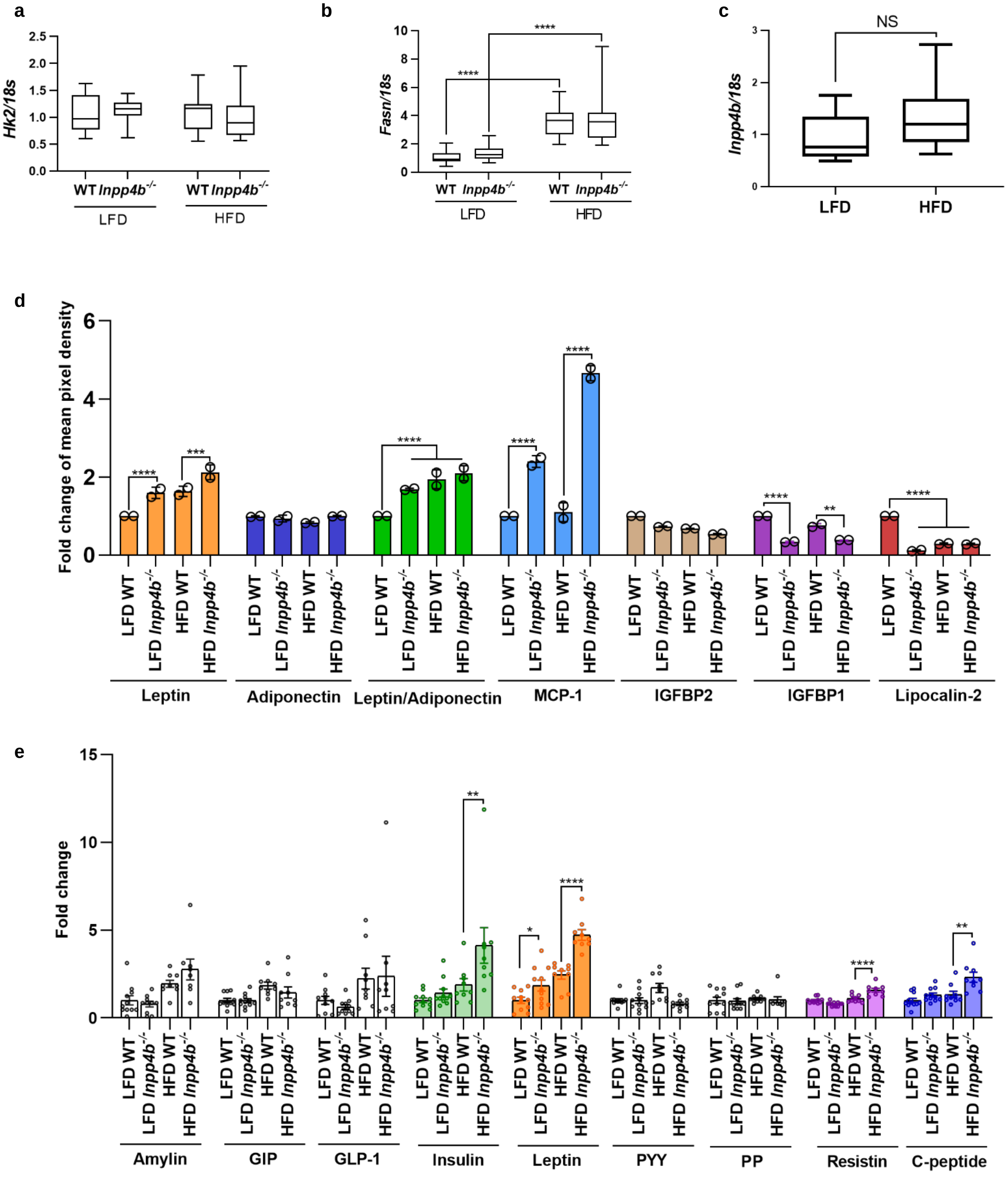

Supplementary figure 4

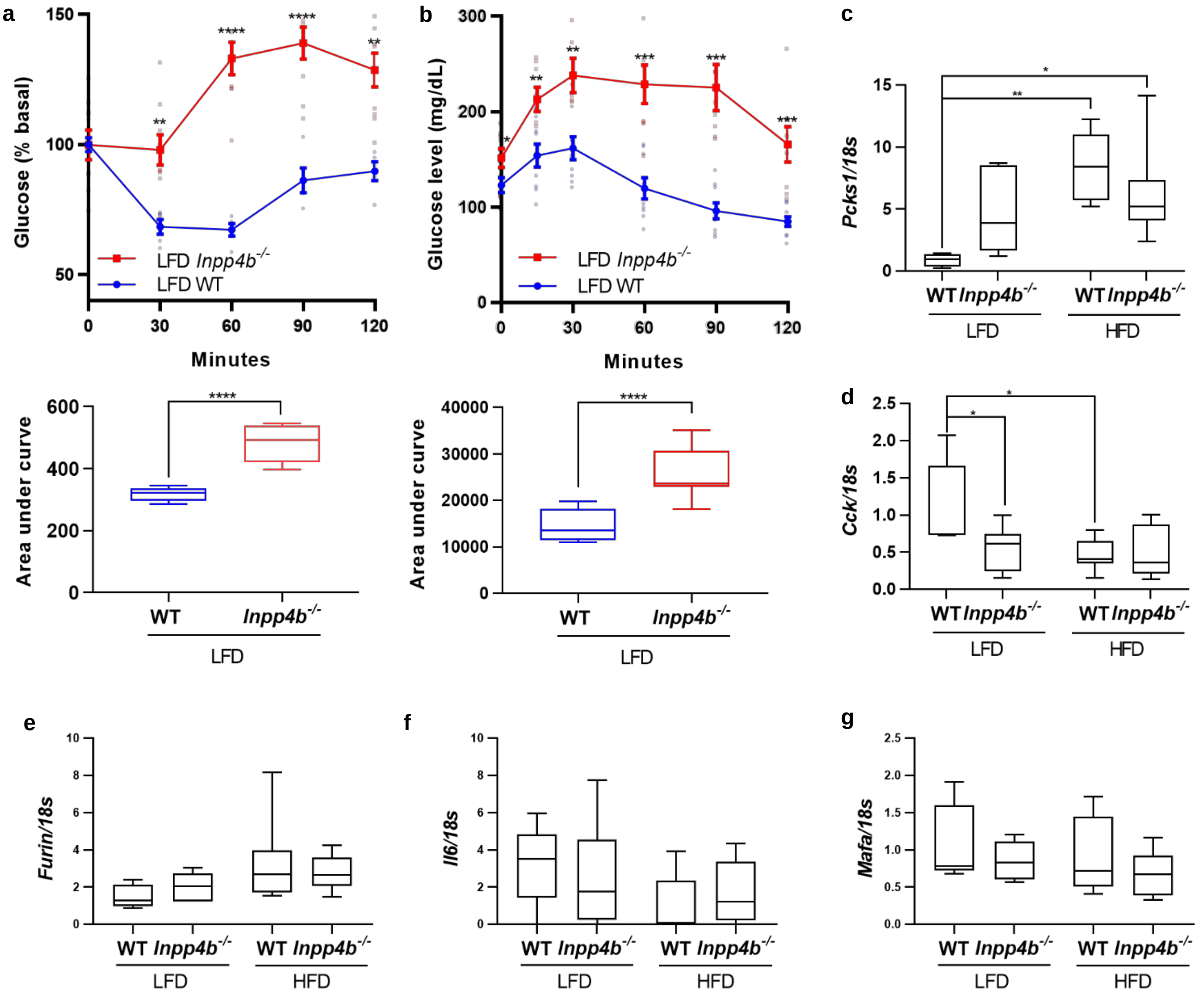

Supplementary figure 5

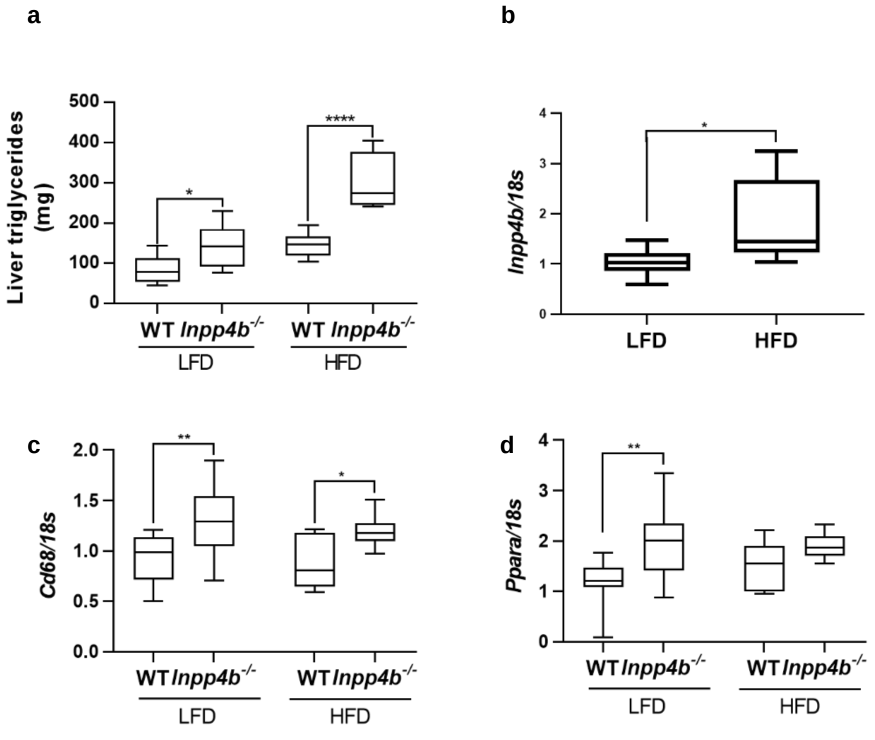

Supplementary figure 6

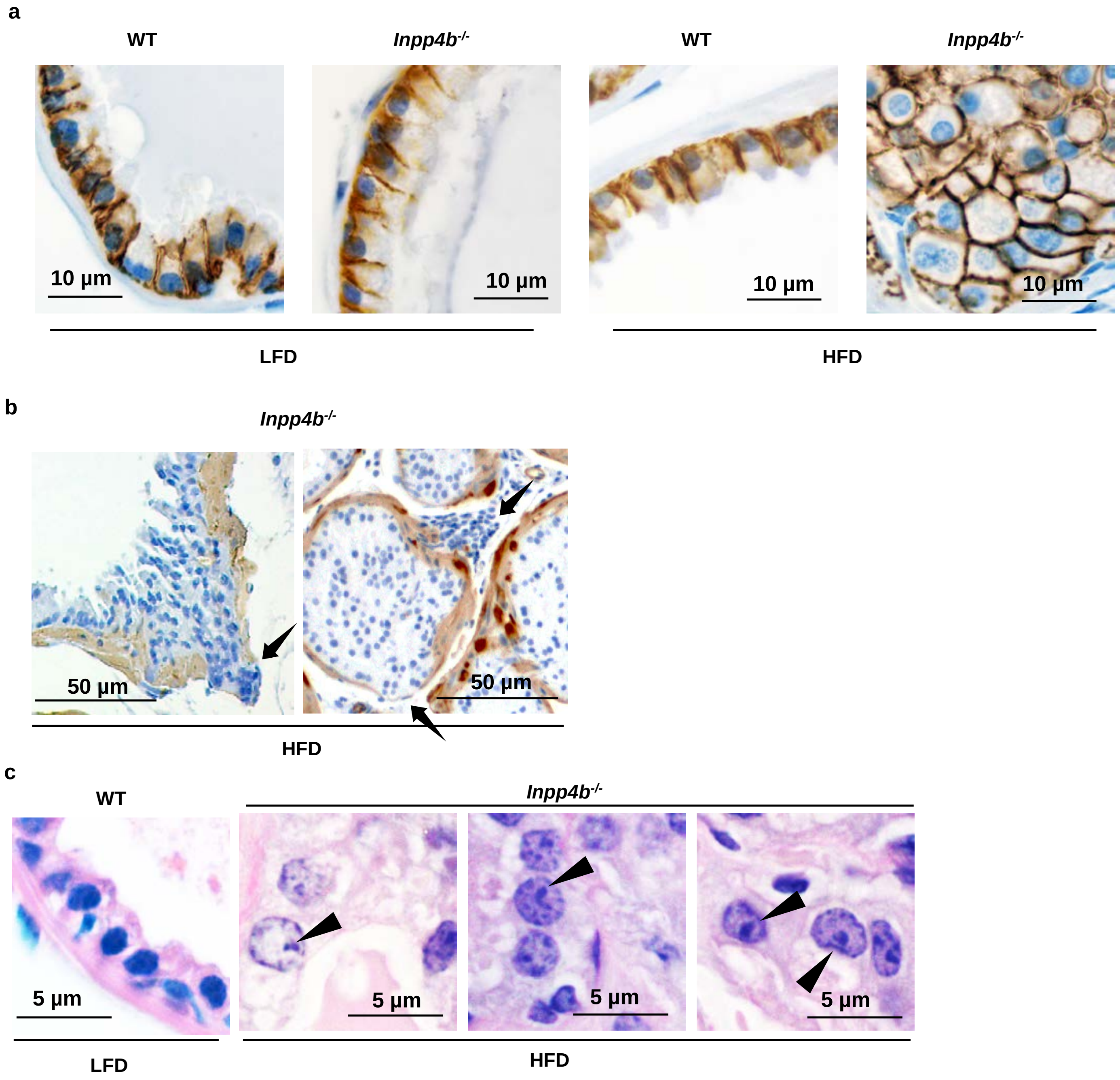

Supplementary figure 7

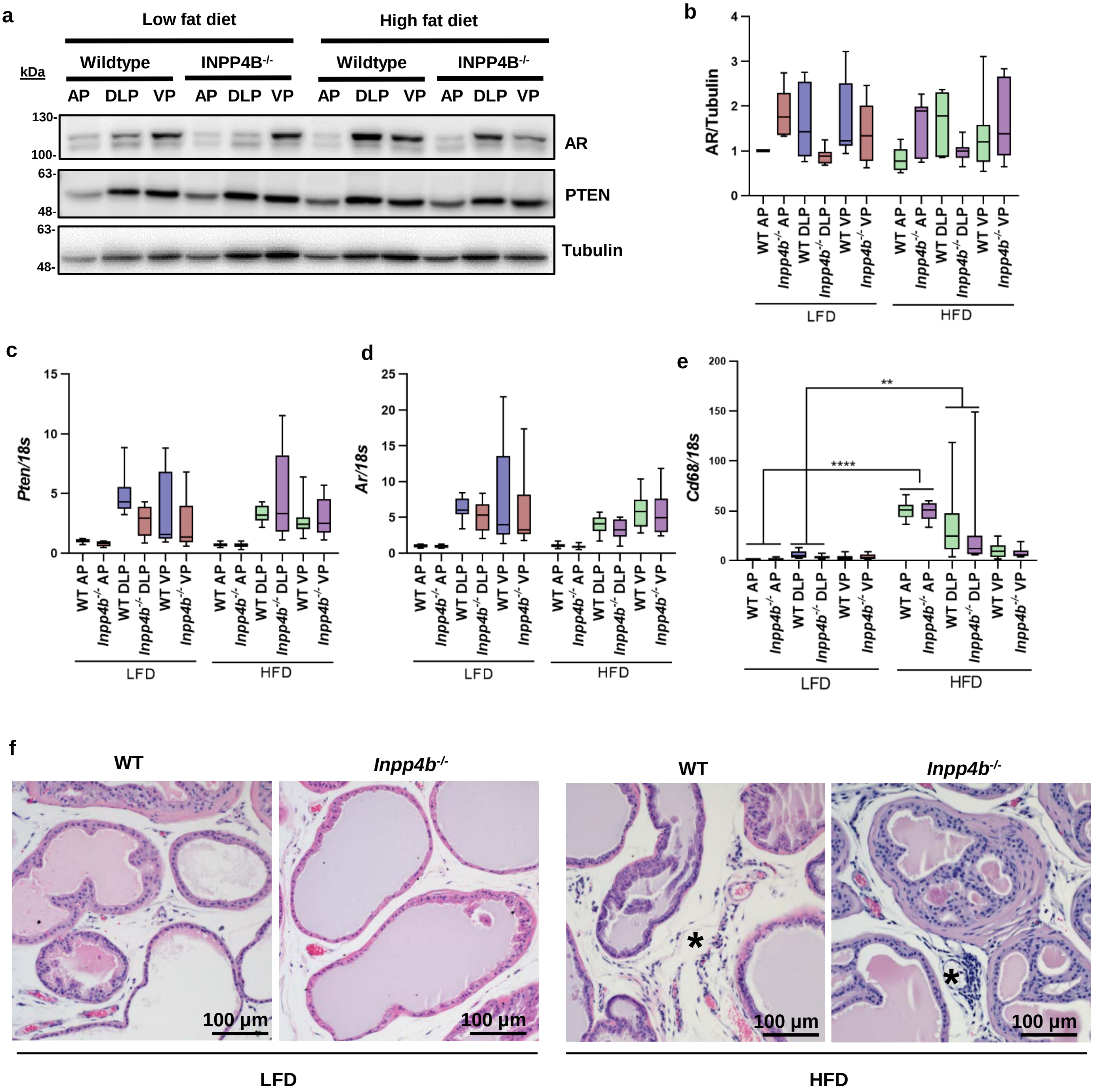

Supplementary figure 8

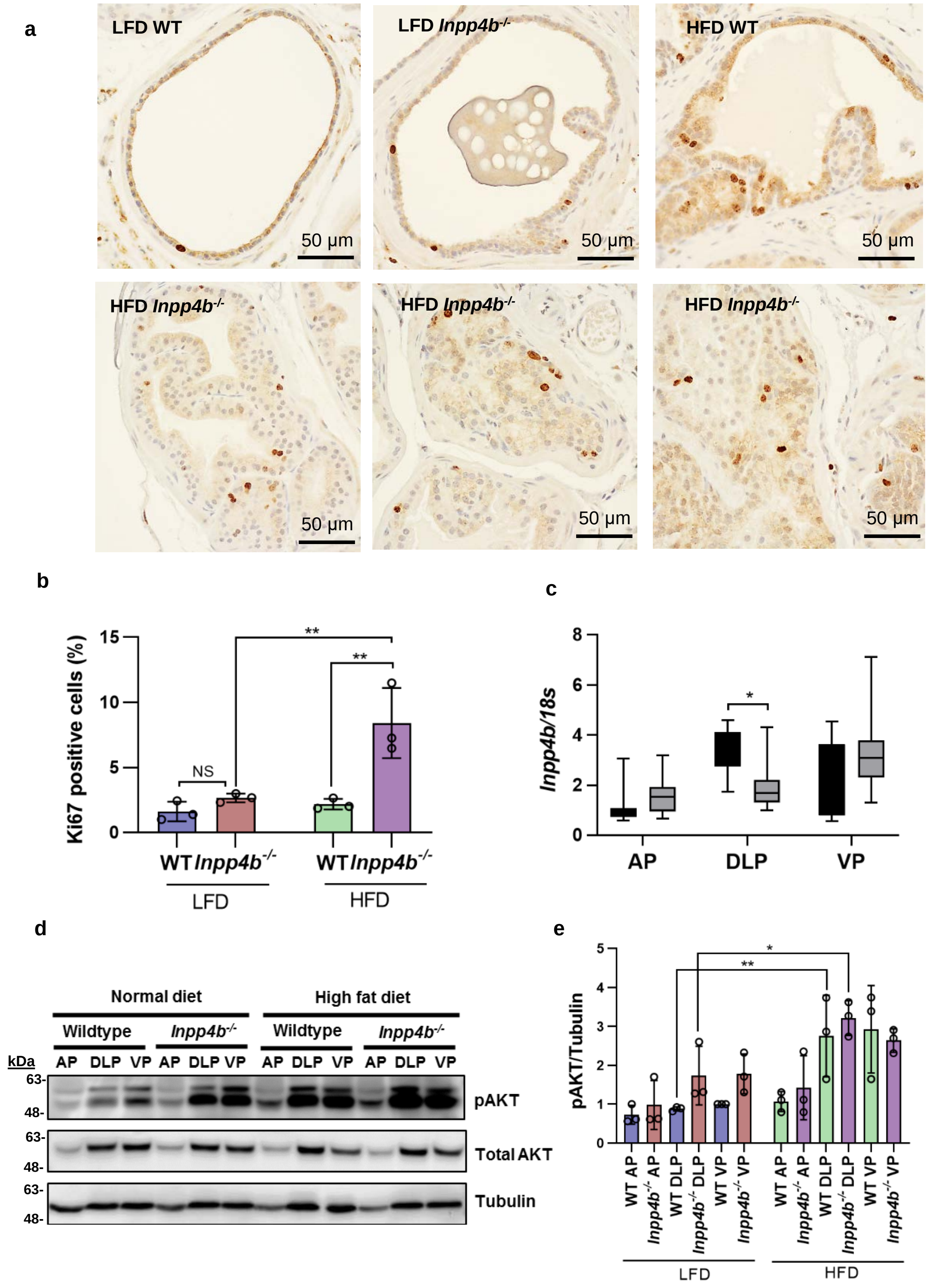

Supplementary figure legends

Supplementary figure 1. Morphometric and metabolic response to HFD and INPP4B loss. (a-c) Body length in centimeters (p= 0.097) (a), blood pressure in millimeter of mercury (mmHg) (b), and heart rate in beats per minute (bpm) (c) were measured for HFD WT (N = 9) and HFD *Inpp4b*$^{-/-}$ mice (N = 10). (d-e) Number 4 mammary glands (d) and epididymal adipose tissues (eWAT) (*p= 0.022) (e) were dissected and their weights compared in LFD WT (N = 13), LFD *Inpp4b*$^{-/-}$ (N = 11), HFD WT (N = 9), and HFD *Inpp4b*$^{-/-}$ (N = 10) groups at 12 weeks of age. (f-g) Average Energy Expenditure kcal/h (f) and Respiratory Exchange Ratio per hour (g) were measured during the last 55 hours of the experiment between WT (N = 6) and *Inpp4b*$^{-/-}$ (N = 8) males (****p<0.0001)

Supplementary figure 2. Histological comparison of adipose tissues. Representative images of the H&E staining of the inguinal, retroperitoneal, mesenteric, epididymal, and brown adipose tissues from LFD WT, HFD WT, LFD *Inpp4b*$^{-/-}$, and HFD *Inpp4b*$^{-/-}$ males. The scale bar is the same in all images and is equal to 50 μm.

Supplementary figure 3. Functional comparison of adipose tissues. (a-b) RNA was extracted from eWAT of LFD WT (N = 11), LFD *Inpp4b*$^{-/-}$ (N = 12), HFD WT (N = 8), and HFD *Inpp4b*$^{-/-}$ (N = 11) mice and analyzed for *Hk2* (a) and *Fasn* (****p<0.0001) (b) expression by qRT-PCR using *18S* as an internal control. (c) The expression levels of *Inpp4b* in eWAT were determined in LFD WT (N = 11) and HFD WT (N = 6) mice by qRT-PCR and normalized to *18S* expression (p = 0.1983). (d) Levels of Leptin, Adiponectin, Leptin/Adiponectin, MCP-1, IGFBP1, IGFBP2, and Lipocalin-2 in the eWAT of LFD WT, LFD *Inpp4b*$^{-/-}$, HFD WT and HFD *Inpp4b*$^{-/-}$ are presented as fold change of relative pixel density. Signal intensity in LFD WT mice was designated as 1. Protein extracts from 5 mice were combined in each group and used for the adipokine array. (e) Serums were obtained from LFD WT (N = 11), LFD *Inpp4b*$^{-/-}$ (N = 11), HFD WT (N = 9), and HFD *Inpp4b*$^{-/-}$ (N = 9) mice and analyzed for amylin, GIP, GLP-1, Insulin, Leptin, PYY, PP, resistin, and C-peptide. (****p<0.0001. Exact p values are provided in the source data)

Supplementary figure 4. Development of hyperglycemia in LFD *Inpp4b*$^{-/-}$ males. (a) Insulin tolerance test and area under the curve in LFD WT (N = 5) and LFD *Inpp4b*$^{-/-}$ (N = 9). (b) Pyruvate tolerance test and area under curve in LFD WT and LFD *Inpp4b*$^{-/-}$ mice (N = 8 per group). Group legends: red line with squares (LFD-WT), blue line with circles (LFD-*Inpp4b* $^{-/-}$). (c-g) RNAs were extracted from mouse pancreas of LFD WT (N = 5), LFD *Inpp4b*$^{-/-}$ (N = 7), HFD WT (N = 8), and HFD *Inpp4b*$^{-/-}$ (N = 8) mice. The expression levels of *Pcsk1* (c), *Cck* (d), *Furin* (e), *Il6* (f), and *Mafa* (g) were assayed by qRT-PCR. (****p<0.0001. Exact p values are provided in the source data).

Supplementary figure 5. Aberrant lipid storage in livers of *Inpp4b*$^{-/-}$ males. (a) The amounts of total triglycerides in liver were measured in LFD WT (N = 8), LFD *Inpp4b*$^{-/-}$ (N = 8), HFD WT (N = 6) and HFD *Inpp4b*$^{-/-}$ (N = 4) mice (*p= 0.0096, ****p<0.0001). (b) RNAs were extracted from mouse livers and the expression of *Inpp4b* was determined in LFD WT (N = 11) and HFD WT (N = 8) mice using qRT-PCR and normalized to *18S* (*p=0.0156). (c-d) The expression of *Cd68* (**p=0.0017 , *p=0.0367) (c) and *Ppara* (**p=0.0012) (d) were analyzed by qRT-PCR in the same animal groups as in (a) with the indicated diets and genotypes (N is the same as panel (a)).

Supplementary figure 6. Histological changes in prostates of HFD *Inpp4b*$^{-/-}$ males. (a) Representative tissue sections from LFD WT, LFD *Inpp4b*$^{-/-}$, HFD WT, and HFD *Inpp4b*$^{-/-}$ prostates were stained for E-cadherin and counterstained with hematoxylin. (b) Representative tissue sections from HFD *Inpp4b*$^{-/-}$prostates stained for SMAα and counterstained with hematoxylin. Arrows indicate discontinuity of fibromuscular layers and neoplastic epithelial cells invasion from prostate gland into prostate stroma. (c) Representative prostate epithelial cell nuclei from LFD WT showing compact architecture and strong nuclear staining (left panel). H&E staining of the atypical hypostained enlarged nuclei with prominent nucleoli frequently observed in hyperplastic sections of the HFD *Inpp4b*$^{-/-}$prostates.

Supplementary figure 7. Prostate-specific levels of AR, PTEN and inflammation in WT and *Inpp4b*$^{-/-}$ males. (a) Protein levels of AR, PTEN, and tubulin in AP, DLP, and VP of LFD WT, LFD *Inpp4b*$^{-/-}$, HFD WT and HFD *Inpp4b*$^{-/-}$ mice were assayed by Western blotting. (b) Quantification of AR protein was done using tissues from five individual mice in the indicated groups; representative Western blot is shown in (a). (c-e) RNAs of AP, DLP and VP of LFD WT (N = 8), LFD *Inpp4b*$^{-/-}$ (N = 9), HFD WT (N = 9), and HFD *Inpp4b*$^{-/-}$ (N = 9) mice were analyzed for *Pten* (c), *Ar* (d) and *Cd68* (****p<0.0001, **p =0.0068 for WT groups, **p=0.0071 for *Inpp4b*$^{-/-}$ groups) (e) expression by qRT-PCR and normalized to *18S*. (f) H&E staining showed immune cell infiltration (labeled with *) in the prostates of WT and knockout males fed with HFD.

Supplementary figure 8. Increased proliferation and Akt signaling in prostates of HFD *Inpp4b*$^{-/-}$ males. (a) Representative prostate tissue sections from LFD WT, LFD *Inpp4b*$^{-/-}$, HFD WT, and HFD *Inpp4b*$^{-/-}$ prostates were stained for Ki67 and counterstained with hematoxylin. (b) Percentage of Ki67 positive cells in LFD WT, LFD *Inpp4b*$^{-/-}$, HFD WT, and HFD *Inpp4b*$^{-/-}$ prostates. (N=3 for each group, NS p= 0.6362, **p=0.014 (HFD groups), **p=0.023 (*Inpp4b*$^{-/-}$ groups)) (c) RNAs were analyzed for *Inpp4b* expression in AP (N = 12), DLP (N = 10) and VP (N = 12) of WT mice fed with LFD or HFD (*p = 0.0409). (d) Protein levels of pAkt, total Akt, and tubulin were assayed in indicated groups by western blotting. (e) Quantification of protein levels of pAkt from the 6 Western blots. The averages were calculated from six individual mice per group. The protein levels were normalized to tubulin and shown as fold change. (*p=0.032, **p= 0.0081)

Figure 2a

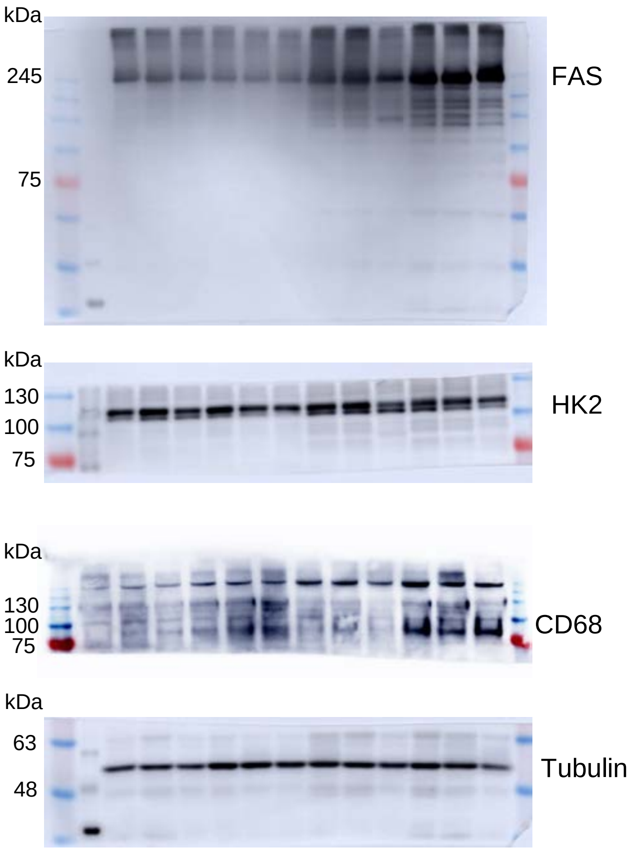

Figure 6b

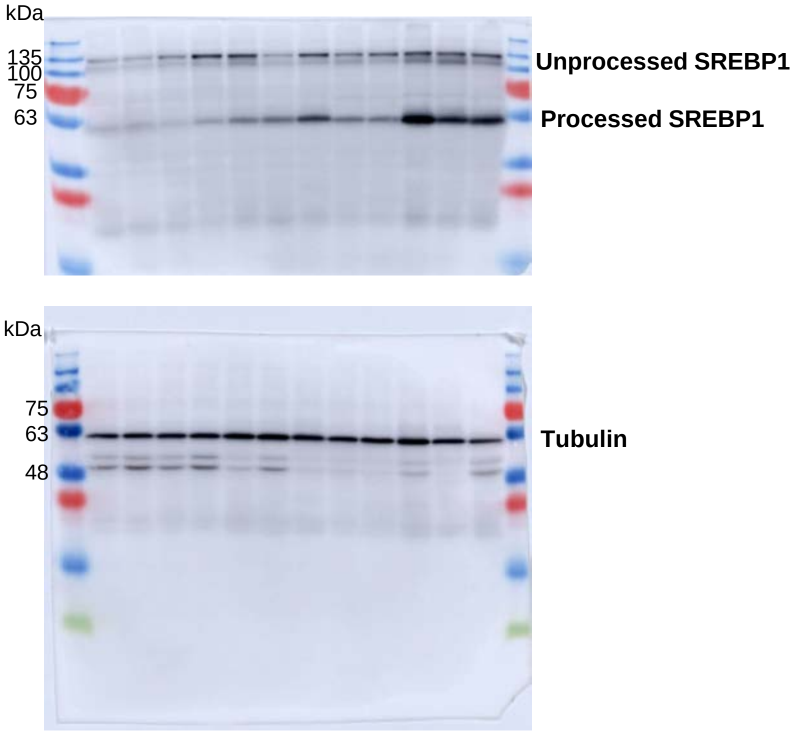

Figure 6d

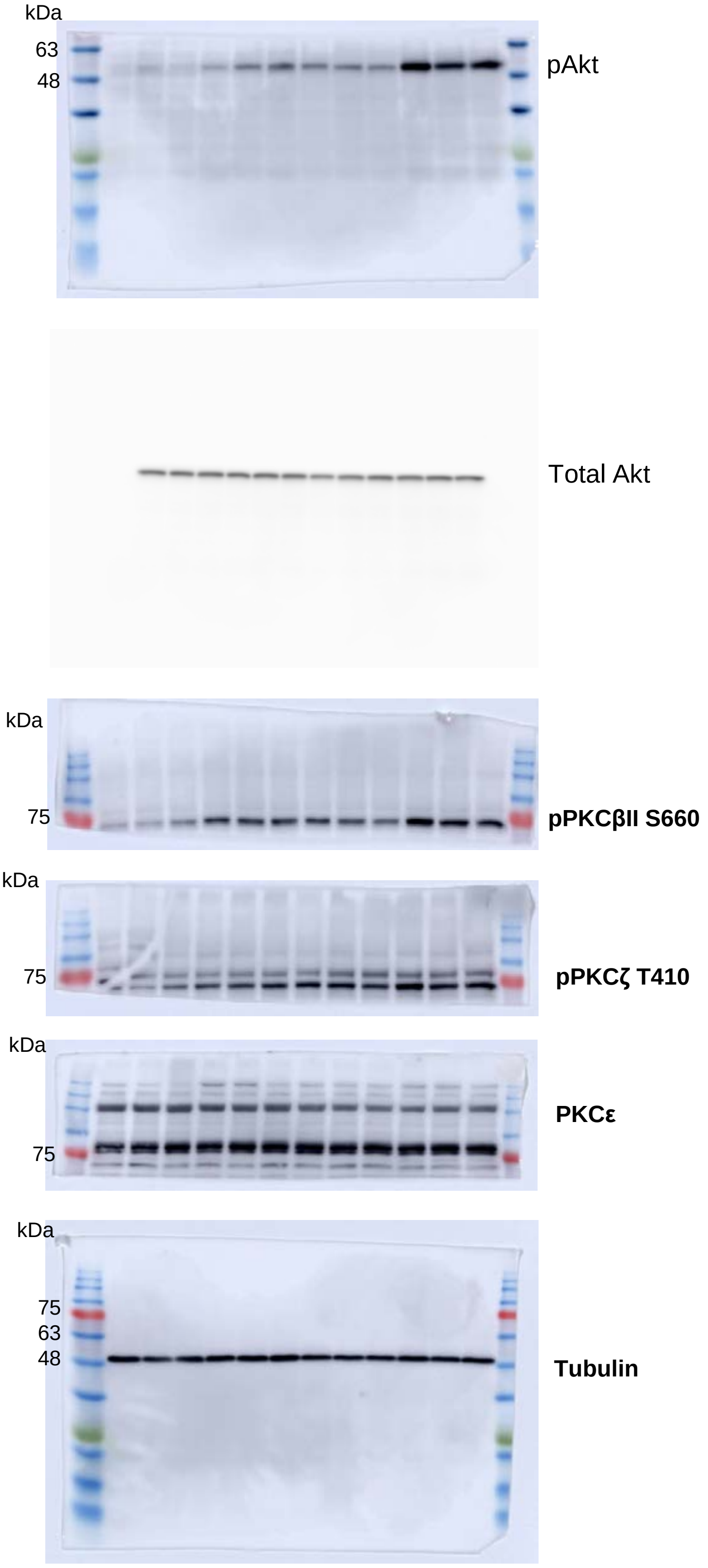

kDa
63
48
pAkt
Total Akt
kDa
75
pPKCβII S660
kDa
75
pPKCζ T410
kDa
75
PKCε
kDa
75
63
48
Tubulin

Figure 6g

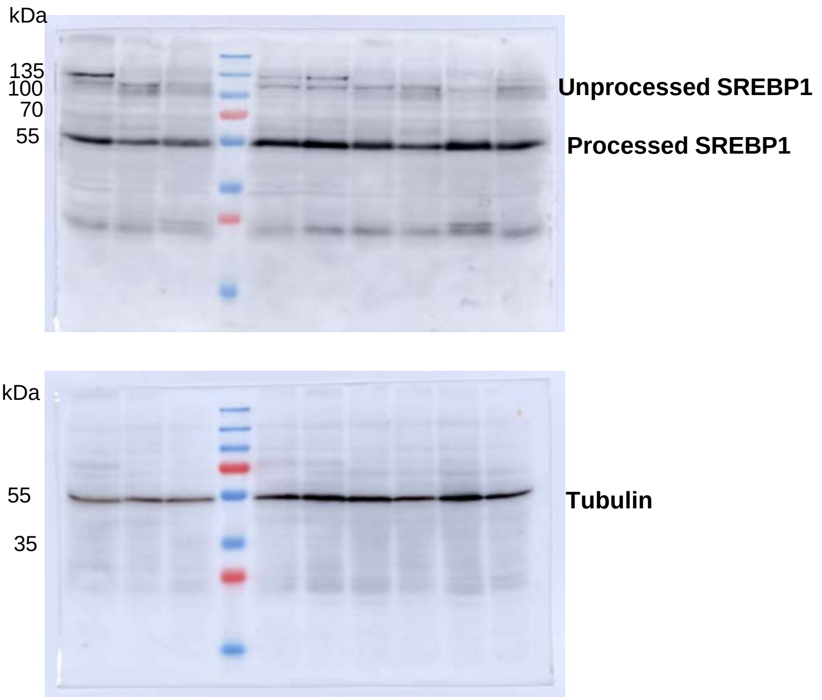

Figure 6j

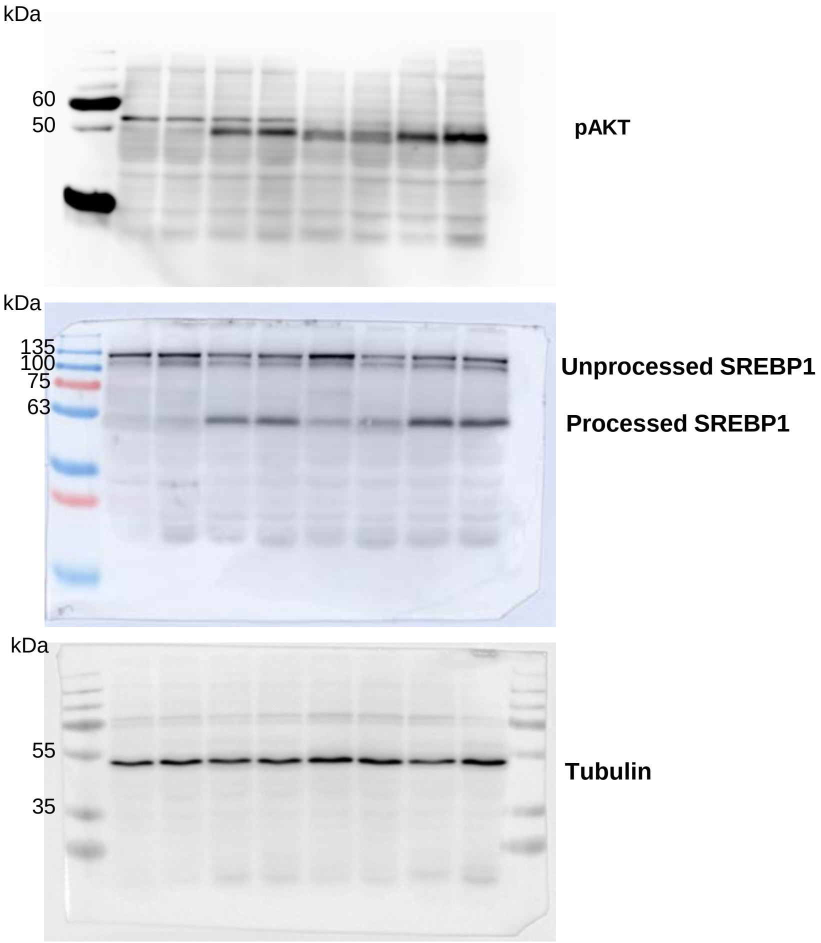

Supplemental figure 7a

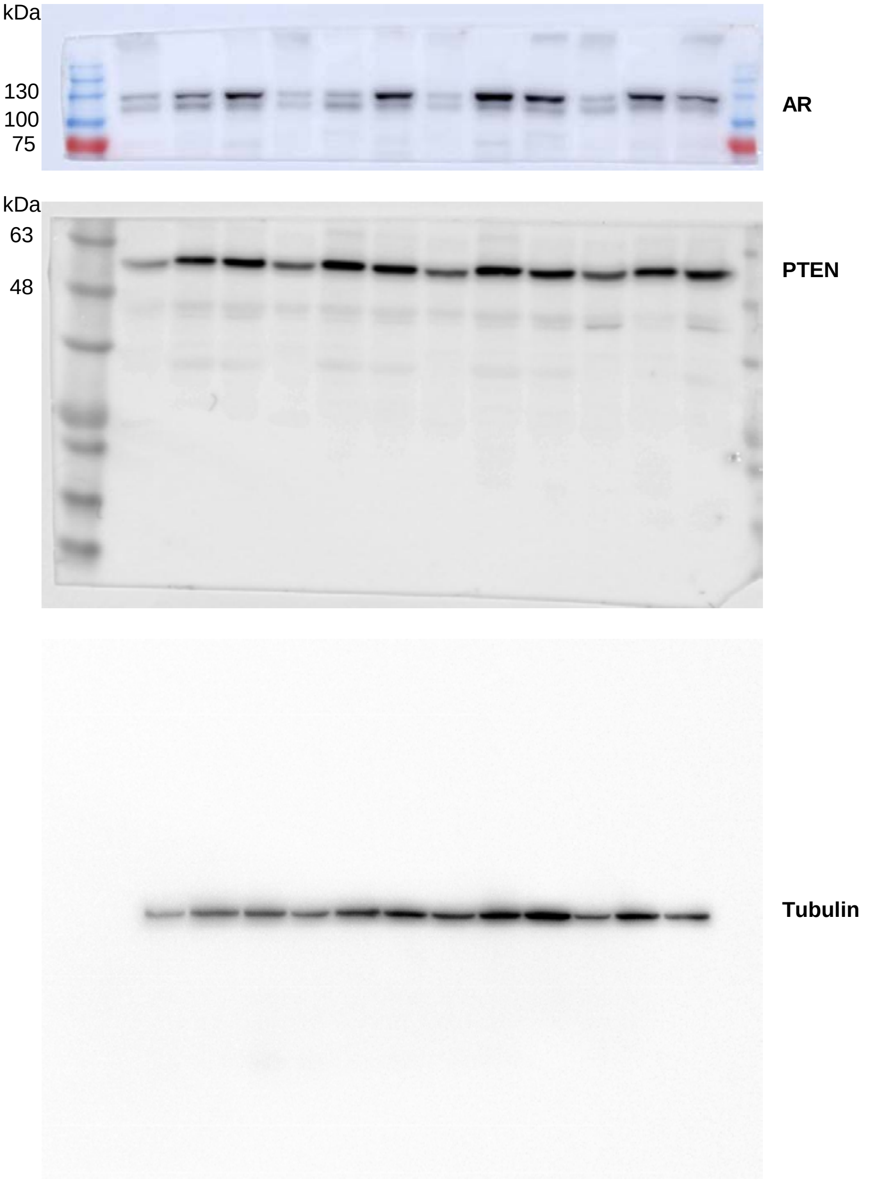

Supplemental figure 8d

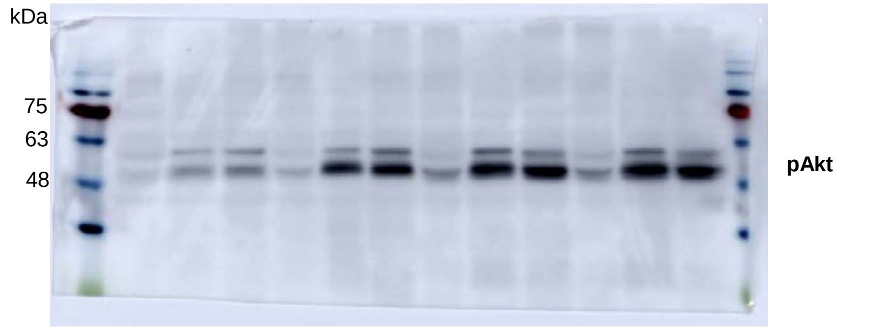

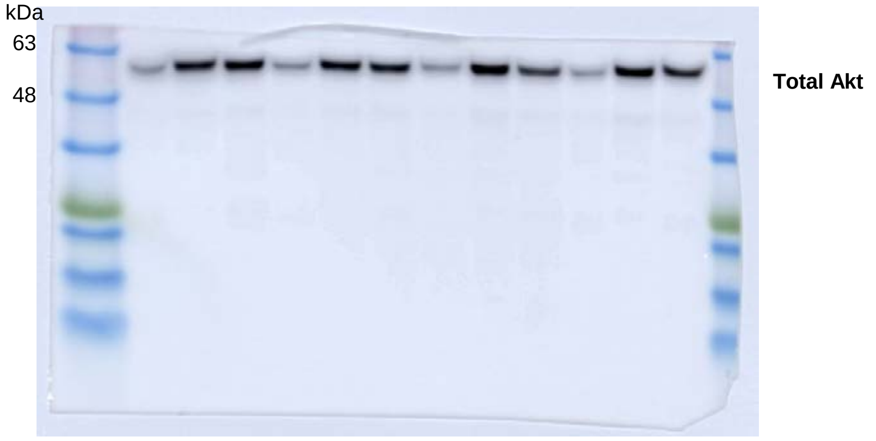

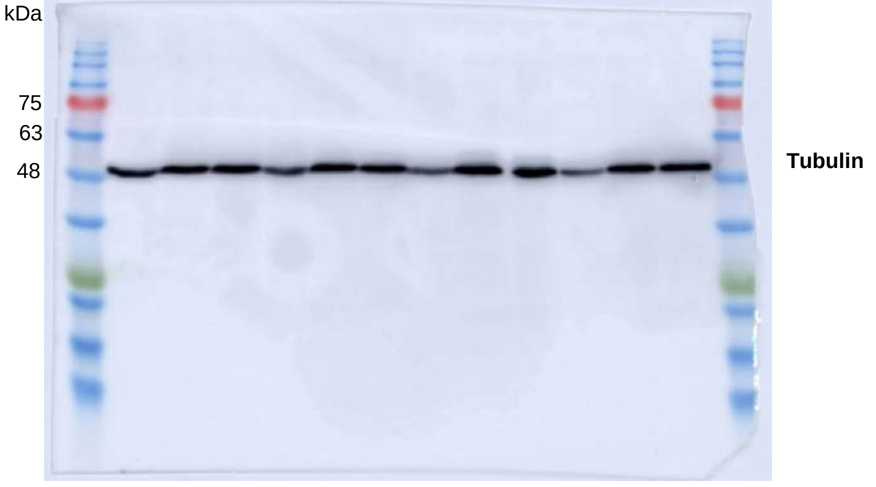

Supplement: Supplementary file 2 — Supplementary Information [file 42003_2021_1940_MOESM2_ESM.pdf]
