# Supplementary material for: INPP4B protects from metabolic syndrome and associated disorders
Source: Commun Biol. 2021 Mar 26;4:416. doi: 10.1038/s42003-021-01940-6 (PMC7998001; doi:10.1038/s42003-021-01940-6)
Supplement: Supplementary file 3 — Description of Additional Supplementary Files [file 42003_2021_1940_MOESM3_ESM.pdf]

Description of Additional Supplementary Files

File Name: Supplementary Data 1

Description: Primers and probes used for gene expression analysis.

File Name: Supplementary Data 2

Description: Source data for Figures 1-7and Supplementary Figures 1,3,4,5,7,8.
