# Supplementary material for: INPP4B protects from metabolic syndrome and associated disorders
Source: Commun Biol. 2021 Mar 26;4:416. doi: 10.1038/s42003-021-01940-6 (PMC7998001; doi:10.1038/s42003-021-01940-6)
Supplement: Supplementary file 6 — Reporting Summary [file 42003_2021_1940_MOESM6_ESM.pdf]

nature research

Corresponding author(s): Irina Agoulnik

Last updated by author(s): 02/10/2021

Reporting Summary

Nature Research wishes to improve the reproducibility of the work that we publish. This form provides structure for consistency and transparency in reporting. For further information on Nature Research policies, see Authors & Referees and the Editorial Policy Checklist .

Statistics

For all statistical analyses, confirm that the following items are present in the figure legend, table legend, main text, or Methods section.

n/a	Confirmed	
☐	☒	The exact sample size (*n*) for each experimental group/condition, given as a discrete number and unit of measurement
☐	☒	A statement on whether measurements were taken from distinct samples or whether the same sample was measured repeatedly
☐	☒	The statistical test(s) used AND whether they are one- or two-sided<br>*Only common tests should be described solely by name; describe more complex techniques in the Methods section.*
☐	☒	A description of all covariates tested
☐	☒	A description of any assumptions or corrections, such as tests of normality and adjustment for multiple comparisons
☐	☒	A full description of the statistical parameters including central tendency (e.g. means) or other basic estimates (e.g. regression coefficient) AND variation (e.g. standard deviation) or associated estimates of uncertainty (e.g. confidence intervals)
☐	☒	For null hypothesis testing, the test statistic (e.g. *F*, *t*, *r*) with confidence intervals, effect sizes, degrees of freedom and *P* value noted<br>*Give P values as exact values whenever suitable.*
☒	☐	For Bayesian analysis, information on the choice of priors and Markov chain Monte Carlo settings
☒	☐	For hierarchical and complex designs, identification of the appropriate level for tests and full reporting of outcomes
☒	☐	Estimates of effect sizes (e.g. Cohen's *d*, Pearson's *r*), indicating how they were calculated

Our web collection on statistics for biologists contains articles on many of the points above.

Software and code

Policy information about availability of computer code

Data collection	For RNA sequencing, the libraries were generated using the NEBNext® Ultra™ RNA Library Prep Kit and used for Illumina 150-bp paired-end sequencing. Quality control assessment was done using Illumina RNA-seq pipeline to estimate genomic coverage, percent alignment and nucleotide quality.
Data analysis	Raw reads were mapped to the reference mouse genome using HISAT2 and STAR software. For the differential analysis of known genes, the reads for each gene aligned by HISAT2 were counted using HTSeq software. Alignment by STAR was run with the option "quantMode TranscriptomeSAM" that allowed counting of reads aligned to each gene. Raw counts from HTSeq and STAR were imported into Bioconductor/R package DESeq2.

For manuscripts utilizing custom algorithms or software that are central to the research but not yet described in published literature, software must be made available to editors/reviewers. We strongly encourage code deposition in a community repository (e.g. GitHub). See the Nature Research guidelines for submitting code & software for further information.

Data

Policy information about availability of data

All manuscripts must include a data availability statement. This statement should provide the following information, where applicable:

- Accession codes, unique identifiers, or web links for publicly available datasets
- A list of figures that have associated raw data
- A description of any restrictions on data availability

RNA-sequencing data have been deposited in NCBI's Sequence Read Archive (SRA) database with accession number GSE134466. The datasets that support the findings of this study are available in (https://www.ncbi.nlm.nih.gov/geo/) with the accession numbers and DOIs: GSE15653 (10.1210/jc.2009-0212) , GSE126848 (10.1152/ajpgi.00358.2018), GSE89091(10.1038/s41374-018-0088-6) and GSE24335(10.1101/gr.112821.110).

Field-specific reporting

Please select the one below that is the best fit for your research. If you are not sure, read the appropriate sections before making your selection.

- [x] Life sciences
- [ ] Behavioural & social sciences
- [ ] Ecological, evolutionary & environmental sciences

For a reference copy of the document with all sections, see nature.com/documents/nr-reporting-summary-flat.pdf

Life sciences study design

All studies must disclose on these points even when the disclosure is negative.

Sample size	The gene expression differences were analyzed using 2 way ANOVA. All data are presented as mean ± SEM. A sample size of 9 in each group has a 95% power to detect an effect size of 0.75 with a significance level (alpha) of 0.05. P-values less than 0.05 were considered statistically significant.<br>For RNA seq, we used the published data set for our estimation of samples size, and it is from AJPGI with comparisons of NAFLD vs CTRL. Prior data (from AJPGI) indicates that the average read count among the prognostic genes is about 400, the average dispersion is 0.1, and the ratio of the geometric mean of normalization factors is 1. We assumed that the total number of genes for testing will be 10,000, and the top 700 genes are prognostic. If the desired minimum fold change is 2.5, we estimated that we will need to study 4 subjects in each group to be able to reject the null hypothesis that the population means of the two groups are equal with probability (power) 0.8 using exact test. The FDR associated with this test of this null hypothesis is 0.1.<br>For western blotting 4-6 samples per variable were used to achieve statistical significance of p<0.05 using 2-way ANOVA.<br>The glucose tolerance test was performed with at least 7 mice in each group. The measurements were analyzed 2-way ANOVA.
Data exclusions	No data were excluded from the analysis
Replication	Three or more independent experiments were used for comparisons of mRNA and protein levels. Two independent groups spaced one year apart were analyzed for each of the following groups: WT/LFD, WT/HFD, Inpp4b-/-/LFD, and Inpp4b-/-/HFD to ensure reproducibility of the phenotypes.
Randomization	Both wild-type or mutant female breeders were distributed randomly between low fat diet or high fat diet groups. All available pups were used for analyses.
Blinding	Investigators for histological analyses were blinded to the animal's group allocation.

Reporting for specific materials, systems and methods

We require information from authors about some types of materials, experimental systems and methods used in many studies. Here, indicate whether each material, system or method listed is relevant to your study. If you are not sure if a list item applies to your research, read the appropriate section before selecting a response.

Materials & experimental systems

n/a	Involved in the study	
	x	Antibodies
x		Eukaryotic cell lines
x		Palaeontology
	x	Animals and other organisms
x		Human research participants
x		Clinical data

Methods

n/a	Involved in the study	
x		ChIP-seq
x		Flow cytometry
x		MRI-based neuroimaging

Antibodies

Antibodies used	α-SMA (#ab5694, AbCam), insulin (#ab181547, AbCam), Ki67 (#RB-1510-P1, Thermo Fisher), phospho-Akt S473 (Cell signaling #4051), total Akt (Cell signaling #4691), phospho-PKCζ T410 (Cell signaling #2060), phospho-PKCβII S660 (Cell signaling #9371), FAS (Cell signaling #3180), HK2 (Cell signaling #2867), Tubulin (Mippore #05-661), PTEN (Cell signailng #9188), Androgen receptor (Mippore #06-680), SREBP1 (Novus NB 600-582), CD68 (Abcam 125212), E-cadherin (Cell Signaling #3195), PKCε (R&D systems AF5134)
Validation	α-SMA (#ab5694, AbCam): Validated in WB, ELISA, IHC, ICC, ICC/IF and tested in Mouse<br>Insulin (#ab181547, AbCam): Walsh SR et al. Type I IFN blockade uncouples immunotherapy-induced antitumor immunity and autoimmune toxicity. J Clin Invest 129:518-530 (2019).<br>Ki67 (#RB-1510-P1, Thermo Fisher):Key G, et al. J Clin Pathol 199346:1080-4<br>phospho-Akt S473 (Cell signaling #4051): target signal induced with an activator and signal is blocked with an inhibitor, and correctly pulled down via IP; #4051 has 463 citations by CiteAb

	total Akt (Cell signaling #4691): validated in-house by IP, IHC, IF, FLOW as shown on the datasheet and website; #4691 has 1747 citations by CiteAb<br>phospho-PKCζ T410 (Cell signaling #2060): validate Western blot showing over-expressed PKC isoforms; phospho-specificity shown in cells treated with (+)TPA or (-)? phosphatase; #2060 has 34 citations by CiteAb<br>phospho-PKCβII S660 (Cell signaling #9371): Western blot with over-expressed PKCβII and no signal detected in PKCβII Ser660/Ala mutant; Western blot analysis of extracts from TPA, Go6983 and/or Bisindolylmaleimide treated cells; #9371 has 104 citations by CiteAb<br>FAS (Cell signaling #3180):validated in-house by WB, IP, IHC, IF as shown on the datasheet and website<br>HK2 (Cell signaling #2867):Blesson CS et al. Sex Dependent Dysregulation of Hepatic Glucose Production in Lean Type 2 Diabetic Rats. Front Endocrinol (Lausanne). 2019 Aug 6;10:538<br>Tubulin (Mippore #05-661):This Anti-β-Tubulin Antibody, clone AA2 is validated for use in WB for the detection of β-Tubulin.<br>PTEN (Cell signailng #9188): validated in-house by WB (high/low expressors of the target), IP, IHC as shown on the datasheet and website<br>Androgen receptor (Mippore #06-680): Asangani, IA et al. Therapeutic targeting of BET bromodomain proteins in castration-resistant prostate cancer. Nature 510 278-82 2014<br>SREBP1 (Novus NB 600-582): Ikenoue T, Terakado Y, Zhu C, Liu X. Establishment and analysis of a novel mouse line carrying a conditional knockin allele of a cancer-specific FBXW7 mutation. Sci Rep. 2018 Jan 31 (WB, Mouse)<br>CD68 (Abcam 125212): Validated in WB, IHC and tested in Mouse, Rat, Human.<br>E-cadherin (Cell Signaling #3195): Validated in WB, IHC, IF, etc in human, mouse and rat samples and was cited 1305 times.<br>PKCε (R&D systems AF5134): Gao, W., Zan, Y., etc. Quercetin ameliorates paclitaxel-induced neuropathic pain by stabilizing mast cells, and subsequently blocking PKCε-dependent activation of TRPV1, Acta Pharmacologica Sinica, 1 September 2016.

Animals and other organisms

Policy information about studies involving animals; ARRIVE guidelines recommended for reporting animal research

Laboratory animals	Species: mus musculus. Strain: FVB/NJ, FVB/Inpp4b-/- . Sex: male. Age: 11-13 weeks old.
Wild animals	No wild animals were used.
Field-collected samples	Not applicable.
Ethics oversight	All protocols were approved by Florida International University Institutional Animal Care and Use Committee.

Note that full information on the approval of the study protocol must also be provided in the manuscript.
